# Tissue-specific range uncertainty estimation in proton therapy

**DOI:** 10.1016/j.phro.2023.100441

**Published:** 2023-04-22

**Authors:** Casper Dueholm Vestergaard, Ludvig Paul Muren, Ulrik Vindelev Elstrøm, Jacob Graversen Johansen, Vicki Trier Taasti

**Affiliations:** a Danish Centre for Particle Therapy, Aarhus University Hospital, Aarhus, Denmark; b Department of Radiation Oncology (MAASTRO), GROW School for Oncology and Reproduction, Maastricht University Medical Centre+, Maastricht, The Netherlands

**Keywords:** Proton range uncertainty, Proton therapy, Treatment planning, Patient specific range uncertainty

## Abstract

**Background and Purpose:**

Proton therapy is sensitive to range uncertainties, which typically are accounted for by margins or robust optimization, based on tissue-independent uncertainties. However, range uncertainties have been shown to depend on the specific tissues traversed. The aim of this study was to investigate the differences between range margins based on stopping power ratio (SPR) uncertainties which were tissue-specific (applied voxel-wise) or fixed (tissue-independent or composite).

**Materials and Methods:**

Uncertainties originating from imaging, computed tomography (CT) number estimation, and SPR estimation were calculated for low-, medium-, and high-density tissues to quantify the tissue-specific SPR uncertainties. Four clinical treatment plans (four different tumor sites) were created and recomputed after applying either tissue-specific or fixed SPR uncertainties. Plans with tissue-specific and fixed uncertainties were compared, based on dose-volume-histogram parameters for both targets and organs-at-risk.

**Results:**

The total SPR uncertainties were 7.0% for low-, 1.0% for medium-, and 1.3% for high-density tissues. Differences between the proton plans with tissue-specific and fixed uncertainties were mainly found in the vicinity of the target. Composite uncertainties were found to capture the tissue-specific uncertainties more accurately than the tissue-independent uncertainties.

**Conclusion:**

Different SPR uncertainties were found for low-, medium-, and high-density tissues indicating that range margins based on tissue-specific uncertainties may be more exact than the standard approach of using tissue-independent uncertainties. Differences between applying tissue-specific and fixed uncertainties were found, however, a fixed uncertainty might still be sufficient, but with a magnitude that depends on the body region.

## 1 Introduction

The favorable depth-dose curve for protons ensures a high dose deposition in a region which can be targeted to the tumor while sparing organs-at-risk (OARs). However, this well-defined dose deposition also represents a major concern since it makes proton therapy sensitive to uncertainties due to e.g. setup, organ motion, and proton range estimation [[1], [2]].

The proton range, and thereby the location of the high-dose region, is directly related to the stopping power ratio (SPR) of the patient’s tissues which typically is estimated from a computed tomography (CT) scan [3]. However, the CT-based estimation of the SPR is subject to inaccuracies originating from differences in interactions of photons and charged particles, which entail uncertainties in the proton range estimation [[2], [4]]. Additional range uncertainties are caused by CT image noise [5] and the beam hardening effect [6], as well as differences between the tissue-surrogates used for calibration and the biological tissues which the estimation method is intended to be used for [3].

Typically, 3.5 % of the proton range is added as a range margin around the tumor or in the robust optimization to ensure full target coverage even in the case of range uncertainties [[1], [7], [8]]. However, protons interact differently with low-density (lung), medium-density (soft), and high-density (bone) tissues, due to their distinct chemical compositions and densities [[9], [10]]. Consequently, the overall range uncertainty will depend on the tissue type and the traditional fixed range uncertainty, e.g. 3.5 %, may therefore not be suitable for all tumor sites [11]. Estimating individual uncertainties for different tissue types enables the use of tissue-specific range uncertainties, which are customized to the specific patient and treatment site [[2], [4], [12], [13], [14]]. This idea was previously explored by Yang et al. [2] who computed a single composite SPR uncertainty based on the tissue-specific SPR uncertainties and the average tissue composition in three treatment sites. Peters et al. [4] also performed a comprehensive analysis of the range uncertainties, as part of their clinical implementation of a dual-energy CT (DECT) based SPR prediction, to determine a composite range uncertainty for three treatment sites. However, both of these studies focused on computing a single composite and treatment-site specific range uncertainty and not on applying the tissue-specific uncertainties directly. The aim of this study was therefore to apply the tissue-specific uncertainties directly on a patient-specific, voxel-wise basis.

## 2 Materials and methods

### 2.1 Tissue classification

**Fig. 1:**
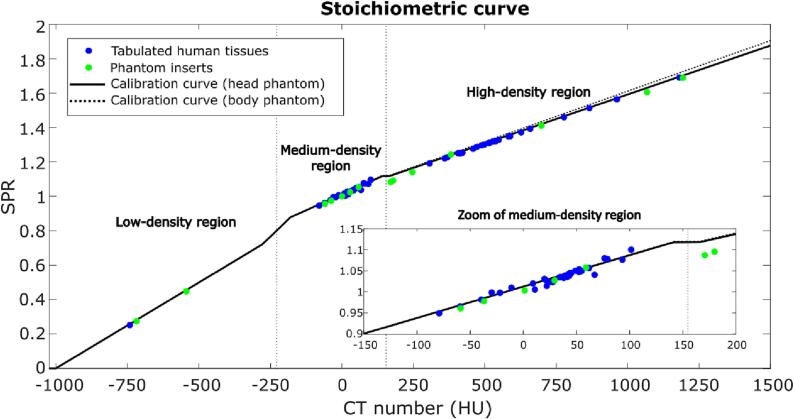
Stoichiometric conversion curves. The solid (dotted) curve represents the stoichiometric curve that has been calibrated using phantom inserts scanned in the head (body) phantom. The blue points represent the CT numbers and SPR values of tabulated human tissues calculated using fitting parameters from the head phantom [[9], [10]]. The green points represent the CT numbers and SPR values of the phantom inserts estimated using fitting parameters from the head phantom (not used for calibration). The inset shows a zoom of the medium-density tissue region. The vertical dotted lines mark the division of the three tissue groups.

Conversion from CT numbers to SPR was performed using the stoichiometric calibration, proposed by Schneider et al. [15]. This conversion curve is based on fitting estimated CT numbers to theoretical SPR values of tabulated human tissues, e.g. as listed by Woodard and White [[9], [10]]. The conversion curve was calibrated using the Advanced Electron Density Phantom (Gammex, Sun Nuclear, Middleton, WI, USA) scanned with a SOMATOM Definition Edge CT scanner (Siemens Healthineers, Forchheim, Germany). Further details on the stoichiometric method and the CT scanner protocol used for phantom scanning are provided in Supplementary Material A. The resultant conversion curves (head and body phantom) are shown in Fig. 1. Tissues were classified into low-, medium-, and high-density tissues based on their CT numbers (H), using the midpoints of the two connecting segments in the conversion curve for the head phantom; voxels with -900HU<H≤-228HU were classified as low-density tissues, voxels with -228HU<H≤154HU were classified as medium-density tissues and vowels with H>154HU were classified as high-density tissues.

### 2.2 SPR uncertainty estimation

The uncertainties in the SPR estimation were divided into five categories according to their origin. The first category of uncertainties concerned the uncertainties arising from the CT image quality (CT imaging uncertainties; $U^{img}$). The second category involved the uncertainties arising from inherent inaccuracies in the CT number estimation for reference human tissues using a parametrization (CT number estimation uncertainty; $U^{H}$). The third category concerned the conversion of CT numbers into SPR values applying the stoichiometric conversion curve for biological tissues (SPR estimation uncertainty; $U^{stoi}$). The remaining two categories of uncertainties were independent of the SPR-estimation method and the CT scanner. These concerned the uncertainty in calculating mean excitation energy ($U^{MEE}$), and the uncertainty due to ignoring the energy dependence of the SPR ($U^{E}$). Both categories have previously been evaluated in the study by Yang et al. [2] and their results were used here. The estimations of the three model-dependent uncertainties are outlined in Supplementary Material B.

The uncertainties were estimated individually for the three tissue groups. Both unsigned (absolute), $U_{uns}$, and relative, $U_{rel}$, SPR uncertainties were estimated:

(1) $$U_{uns}=\alpha \cdot \left|\sigma_{H}\right|$$

(2) $$U_{rel}=\frac{U_{uns}}{S}\cdot 100\%$$

where α is the slope of the line segment of the conversion curve for the specific tissue group (low-, medium- and high-density materials), $|\sigma_{H}|$ is the unsigned variation in CT number, and S is the estimated SPR value.

Assuming the five categories of uncertainties to be independent for simplicity, as done in previous studies [[2], [13]], the total unsigned/relative uncertainties were obtained by adding the five different uncertainties in quadrature for each tissue group according to the Guide to the expression of Uncertainty in Measurement [16] ( see Supplementary Material B):

(3) $$U_{uns/rel}^{tot}=\sqrt{{\left(U_{uns/rel}^{img}\right)}^{2}+{\left(U_{uns/rel}^{H}\right)}^{2}+{\left(U_{uns/rel}^{stoi}\right)}^{2}+{\left(U_{uns/rel}^{MEE}\right)}^{2}+{\left(U_{uns/rel}^{E}\right)}^{2}}$$

The unsigned SPR uncertainties of the two model-independent uncertainties were estimated from the relative uncertainties using the mean SPR values of the phantom inserts belonging to the three tissue groups (0.4, 1.0 and 1.3 for low-, medium- and high-density tissues, respectively).

Composite range uncertainties (integrated over the beam path) of different treatment cases were obtained by weighting the total SPR uncertainties (voxel level) with respect to the relative percentages of low-, medium- and high-density tissues in the beam paths:

(4) $$SPR_{comp}=U_{W}^{tot,L}*\omega_{L}+U_{W}^{tot,M}*\omega_{M}+U_{W}^{tot,H}*\omega_{H}$$

(5) $$R_{comp}=1.5*SPR_{comp}$$

where ${{SPR}_{comp}/R}_{comp}$ are the composite SPR/range uncertainties, $U_{W}^{L}$, $U_{W}^{M}$ and $U_{W}^{H}$ are the total SPR uncertainties (relative to SPR of water) for the low-, medium- and high-density tissue group respectively, and $\omega_{L}$, $\omega_{M}$, and $\omega_{H}$ are the relative percentages of low-, medium- and high-density tissues in the beam path. The relative percentages were calculated from the number of voxels classified as low-/medium-/high-density material in the beam path divided by the total number of voxels in the beam path for each treatment site (see Table C1 in Supplementary Material C). Following previous studies, a SPR uncertainty level of 1.5σ was chosen as representative for a range uncertainty on a 2σ level [[1], [4], [12]]. All SPR uncertainties will in the following be reported on a 1σ level, while all range uncertainties will be reported on a 1.5σ level.

### 2.3 Evaluation

The tissue-specific SPR uncertainties (Eq. (3)) were assumed to provide the best estimate of the actual SPR uncertainties of a specific treatment. Hence, the aim of the evaluation was to examine if a single fixed (tissue-independent or composite) uncertainty was sufficient to capture the tissue-specific SPR uncertainties or if tissue-specific uncertainties were required to accurately describe the range uncertainties of the treatment.

**Fig. 2:**
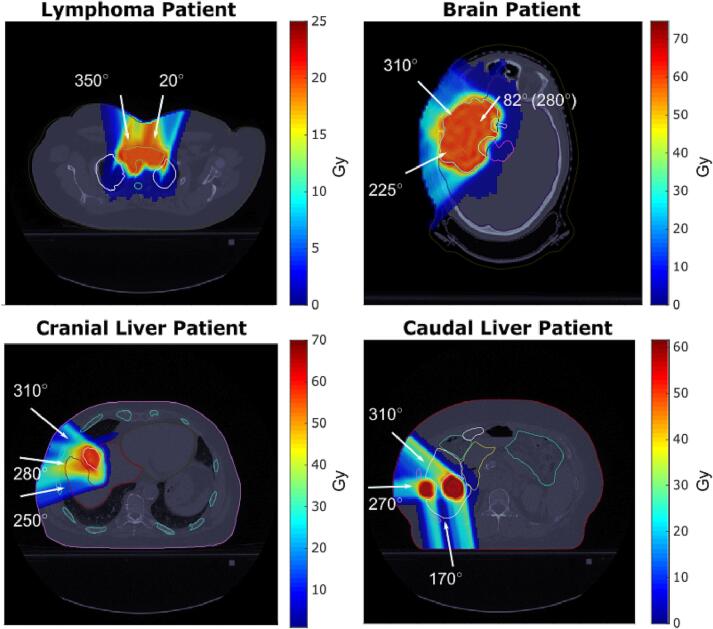
Dose distributions for the four patients. The white arrows indicate the beam angles. For the brain patient, the non-zero couch angle is given in parentheses.

The tissue-specific SPR uncertainties were evaluated using proton plans of four patients treated at our institution: a brain, a lymphoma, and two liver cancer patients (Fig. 2). Two liver cancer patients were included due to different amounts of low-density tissue within the beam path; one with tumor located close to the diaphragm (cranial liver cancer patient) and one with abdominally located tumor (caudal liver cancer patient; Table C1 in Supplementary Material C). Permission to use patient data from our institution was issued by the local hospital authorities. Proton treatment plans were optimized in matRad [[17], [18]] using the prescribed dose, number of fractions, and beam and couch angles from the clinically approved and applied treatment plan (Table C1 in Supplementary Material C). The plan optimization was based on the objectives and constraints from the clinical plan but was intentionally performed without robust optimization to clearly see the effect of using different types of uncertainties.

To evaluate the difference between the use of tissue-specific and fixed SPR uncertainties, the proton plan for each patient was recalculated on perturbed SPR maps created by adding/subtracting the different types of uncertainties to/from the nominal SPR map. The tissue-specific uncertainties were applied voxel-wise according to the CT number, while for the fixed uncertainties the same uncertainty was added to all voxels. Eleven different fixed uncertainty plans were generated: ten with tissue-independent uncertainties in the range [0.5 %;5.0 %] in steps of 0.5 %, and one with the composite SPR uncertainty (Eq. (4)) for the treatment site.

**Fig. 3:**
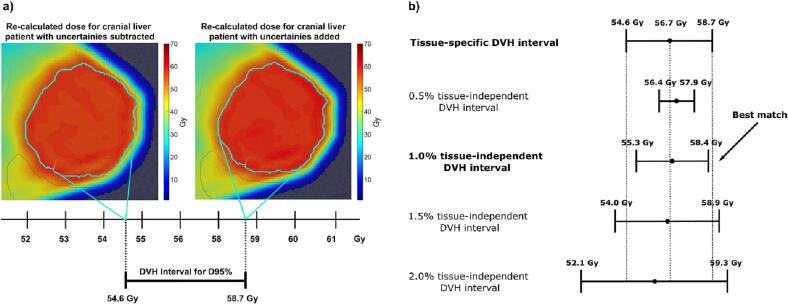
Example of how the best-matching tissue-independent SPR uncertainty was determined. (a) Illustration of how the tissue-specific DVH interval was calculated for D95% of the CTV (delineated in light blue). (b) Illustration of how the tissue-independent DVH intervals were compared to tissue-specific DVH interval. The tissue-independent DVH intervals were compared to the tissue-specific DVH interval in terms of deviations from the midpoint (black circles) and the length of the tissue-specific DVH interval (dotted lines; Eq. (5)).

The differences in the dose-volume-histogram (DVH) parameters of the proton plan with one type of SPR uncertainties added and subtracted spanned a so-called DVH interval (Fig. 3a). The DVH intervals obtained from the proton plans with the fixed uncertainties (tissue-independent or composite DVH intervals) were compared to the DVH interval obtained from the proton plan with tissue-specific uncertainties (tissue-specific DVH interval). The best matching tissue-independent uncertainty ([0.5 %;5.0 %]) was determined as the one resulting in the highest degree of overlap between the tissue-independent and tissue-specific DVH intervals (Fig. 3b). Hence, the best matching tissue-independent SPR uncertainty was found as the one that minimized the difference ΔDI defined as:

(6) $$\begin{array}{c} \Delta DI=\left|L^{TS}-L^{TI}\right|+\left|M^{TS}-M^{TI}\right|\\ \;=\left|\left({DVH}_{add}^{TS}-{DVH}_{sub}^{TS}\right)-\left({DVH}_{add}^{TI}-{DVH}_{sub}^{TI}\right)\right|\\ \;+\left|\left({DVH}_{add}^{TS}+{DVH}_{sub}^{TS}\right)/2-\left({DVH}_{add}^{TI}+{DVH}_{sub}^{TI}\right)/2\right| \end{array}$$

where $L^{TS/TI}$ and $M^{TS/TI}$ are the length and midpoints of the tissue-specific and tissue-independent DVH intervals, respectively, and ${DVH}_{add}^{TS/TI}$ and ${DVH}_{sub}^{TS/TI}$ are the DVH values of the proton plans with tissue-specific/tissue-independent SPR uncertainties added and subtracted, respectively. The DVH intervals were compared for each of the clinically used DVH parameters for the clinical target volume (CTV) and the OARs as well as for ring structures created at the border of the CTV structure (intruding 5 mm into the CTV (CTV_in_) or extruding 5 mm out of the CTV (CTV_out_)), since the biggest influence of SPR uncertainties was assumed to be seen at the border of the target. The maximum dose was quantified by D0.1 cc (D0.03 cc) for the liver/lymphoma (brain) cancer patients, whereas the minimum dose, D_min_, was computed as the smallest voxel value.

It was assessed if a single tissue-independent SPR uncertainty accurately could capture the tissue-specific SPR uncertainties for all relevant DVH parameters for each treatment site. If this was not the case, the tissue-independent SPR uncertainty that provided the smallest overall difference (sum of the unsigned differences for all considered DVH parameters of the treatment site) between the tissue-specific and tissue-independent DVH intervals was chosen for further evaluation.

## 3 Results

### 3.1 Uncertainty analysis

The dominant uncertainty factor for the low-density tissues was the CT imaging uncertainty, while for the medium- and high-density tissues it was the SPR estimation uncertainty (Table 1). The three contributions to the CT imaging uncertainties had a similar influence on medium-density tissues (0.3 %–0.4 %), whereas the patient size was the dominant factor for both low- and high-density tissues (4.3 % and 0.6 %, respectively). For the CT number estimation uncertainty, the largest difference between the estimated and measured CT numbers was found for low-density tissues (4.7 % vs 0.2 %–0.3 % for medium- and high-density tissues). For the SPR estimation uncertainty, it was found that medium- and high-density tissues were especially influenced by change in the mass density (0.7 %–0.8 %).

**Table 1:** Total relative and unsigned SPR uncertainties ($U_{rel/uns};$ Eq. (1), (2), (3)) for the three tissue groups (on a 1σ level). For the CT imaging uncertainties, both the individual contributions (noise, patient size and tissue positioning) as well as the total CT imaging uncertainties are given.

**Total SPR Uncertainties**			
	**Low-density** Relative (unsigned)	**Medium-density** Relative (unsigned)	**High-density** Relative (unsigned)
Total CT Imaging uncertainties	5.2 % (0.020)	0.6 % (0.006)	0.7 % (0.009)
• Noise	1.3 % (0.005)	0.3 % (0.004)	0.3 % (0.004)
• Patient Size	4.3 % (0.016)	0.4 % (0.004)	0.6 % (0.008)
• Tissue Positioning	2.6 % (0.009)	0.3 % (0.004)	0.2 % (0.002)
CT Number Estimation Uncertainties	4.7 % (0.017)	0.2 % (0.002)	0.3 % (0.004)
SPR Estimation Uncertainties	0.2 % (0.001)	0.7 % (0.008)	0.8 % (0.010)
Uncertainties of mean excitation energy	0.2 % (0.001)	0.2 % (0.002)	0.6 % (0.008)
Uncertainties due to energy dependence of SPR	0.2 % (0.001)	0.2 % (0.002)	0.4 % (0.005)
**Total SPR Uncertainty (**1σ**)**	**7.0 % (0.025)**	**1.0 % (0.011)**	**1.3 % (0.017)**

The composite SPR/range uncertainties (1σ/1.5σ; Eq. (4), (5)) over the beam paths were found to be 1.3 %/2.0 % for the lymphoma cancer patient, 1.2 %/1.8 % for the cranial liver cancer patient, 1.1 %/1.6 % for the caudal liver cancer patient and 1.3 %/1.9 % for the brain cancer patient.

### 3.2 DVH-based evaluation

**Fig. 4:**
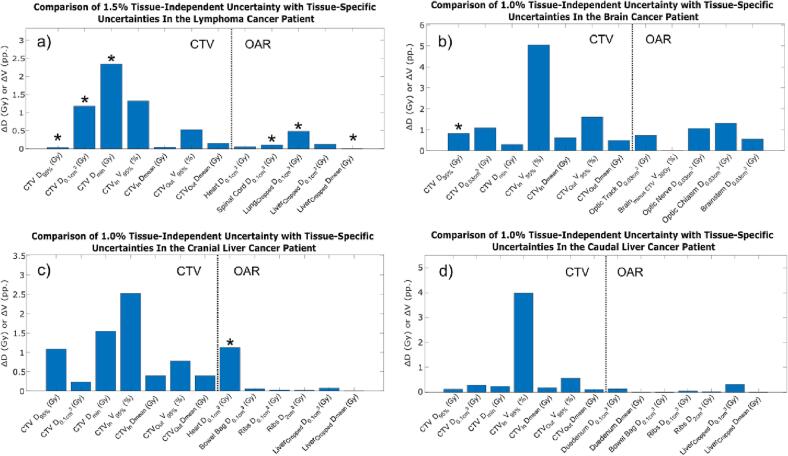
Difference between the DVH intervals, ΔDI (Eq. (6)), of DVH parameters for the proton plan re-calculated with tissue-specific and tissue-independent SPR uncertainties for a) the lymphoma; b) the brain; c) the cranial liver; and d) the caudal liver cancer patient. The dotted line separates DVH parameters for the CTV and the OARs. CTV_in_ and CTV_out_ denote the 5 mm ring structures inside and outside the CTV, respectively, while lung_cropped_ and liver_cropped_ denote the lung/liver cropped to the CTV + δ (δ = 7 mm for the liver patients, δ = 5 mm for the lymphoma patient). Asterisks indicate DVH parameters where another tissue-independent uncertainty was better fitting than the overall best-fitting tissue-independent uncertainty.

When comparing the tissue-specific uncertainties with the tissue-independent uncertainties, it was found that a single tissue-independent uncertainty of 1.0 % provided the best fit for all DVH parameters for the caudal liver cancer patient (Fig. 4). For the remaining three patients, different best-fitting tissue-independent SPR uncertainties were found for the different DVH parameters. However, for most of the DVH parameters, the absolute differences between the tissue-independent and tissue-specific DVH intervals increased only slightly when applying a single tissue-independent SPR uncertainty for all DVH parameters for each treatment site. The single best-fitting tissue-independent SPR uncertainties were found to be 1.5 % for the lymphoma cancer patient and 1.0 % for the brain and the cranial liver cancer patients. When comparing the re-calculated proton plans with tissue-specific uncertainties and the best-fitting tissue-independent uncertainties, dose differences were mainly found in the vicinity of the CTV.

**Fig. 5:**
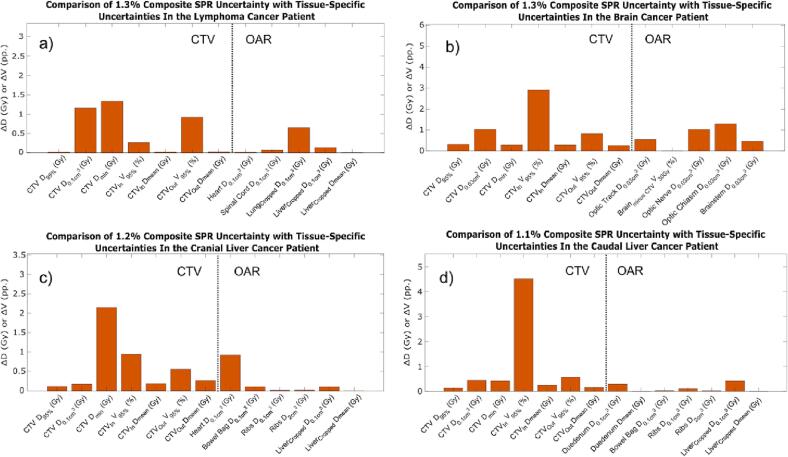
Difference between the DVH intervals, ΔDI (Eq. (6)), of DVH parameters for the proton plan re-calculated with tissue-specific and composite SPR uncertainties for a) the lymphoma; b) the brain; c) the cranial liver; and d) the caudal liver cancer patient. The dotted line separates DVH parameters for the CTV and the OARs. CTV_in_ and CTV_out_ denote the 5 mm ring structures inside and outside the CTV, respectively, while lung_cropped_ and liver_cropped_ denote the lung/liver cropped to the CTV + δ (δ = 7 mm for the liver patients, δ = 5 mm for the lymphoma patient).

When comparing the tissue-specific DVH intervals to the composite DVH intervals instead of the tissue-independent ones, slightly smaller differences were generally found (compare Fig. 4, Fig. 5). For the lymphoma, brain and cranial liver cancer patient the absolute differences were similar or smaller with using composite SPR uncertainties instead of the tissue-independent ones, expect for the V_95%_ of the CTV_out_ for the lymphoma cancer patient and the D_min_ of the CTV for the cranial liver cancer patient. For the caudal liver cancer patient, the absolute differences were found to be similar or larger when using the composite SPR uncertainties instead of the tissue-independent ones, notably for the V_95%_ of the CTV_in_.

## 4 Discussion

This study investigated the feasibility of using tissue-specific SPR uncertainties on a voxel-wise basis as an alternative to using a single fixed (tissue-independent or composite) SPR uncertainty for all voxels. The use of tissue-specific uncertainties was evaluated by comparing proton plans re-computed with tissue-specific and fixed uncertainties applied. Absolute differences between the tissue-specific and fixed SPR uncertainties were overall smallest when using the composite SPR uncertainties for three out of four patients. However, notable differences were found for some DVH parameters regardless of the choice of fixed SPR uncertainty, indicating that there could be a gain from applying the tissue-specific uncertainties on a voxel-wise basis instead of using a single fixed uncertainty.

Our SPR uncertainty categorization was inspired by the study performed by Yang et al. [2]. This group initially performed the analysis for SECT and has later extended their study to monoenergetic CT (MonoCT) which was used in this study [13]. In their analyses, they found the smallest (0.9 %–1.6 %) and largest (3.2 %–5.0 %) uncertainties for medium- and low-density tissues, respectively. They found that the largest contributions to the total SPR uncertainties came from CT imaging and CT number estimation uncertainties for low-density tissues, SPR estimation uncertainties for medium-density tissues, and CT imaging uncertainties for high-density tissues. These findings are in good agreement with our results (Table 1). In good consistency with their studies, we found that the low-density tissue group yielded very large relative uncertainties compared to the other two tissue groups (7.0 % vs 1.0 %–1.3 %). However, the overall effect of the SPR uncertainty for low-density tissues is small compared to the relative uncertainties of the other tissue groups as the SPR values which correspond to these large relative uncertainties are small, making the absolute SPR uncertainty comparable to the other tissue groups (Table 1).

In accordance with other studies, we assumed for simplicity that each individual uncertainty could be considered independently [[2], [13]]. However, the CT imaging uncertainties may have affected the estimation of the CT number and SPR estimation uncertainties. While the impact of CT imaging noise may be small, as positive and negative variations could compensate each other (assuming the noise to be symmetrical), the impact from the beam hardening effect should be included in a more thorough uncertainty analysis [4].

Recently Peters et al. [4] evaluated the range uncertainties in low-, medium- and high-density tissues for DECT. Compared to our study, this group considered a much more extensive and clinically robust evaluation of the range uncertainties. Still, the group reported smaller absolute SPR uncertainties compared to our findings (1.3 %/1.3 %/1.6 % vs 2.5 %/1.0 %/1.7 % for low-/medium-/high-density tissues) as well as a smaller composite range uncertainty (on a 1.5σ level) for brain cancer patients (1.7 % vs 1.9 %; note only one brain cancer patient was included in our study). Part of the reason for their lower uncertainties may be their full DECT-based calibration method. In agreement with our results, they found the impact of patient geometry differing from the calibration condition to be the dominant contribution to the range uncertainty. Thus, further inclusion of the patient size in the SPR calibration could be promising for further reducing the range uncertainties.

It should be noted that while the SPR uncertainty analysis was performed on MonoCT images, only the brain patient had a MonoCT-based treatment plan while the remaining patients had SECT-based treatment plans. Thus, it can be hypothesized that the tissue-specific SPR uncertainties could have been different for the three other patients. Wohlfahrt et al. [6] found that using MonoCT with the MonoPlus algorithm instead of 120 kVp SECT resulted in a reduction in image noise and beam-hardening. Thus, slightly higher CT imaging uncertainties could be expected if the analysis had been performed on SECT. This is consistent with results from Yang et al. [2] and Je et al. [13], where all three model-dependent uncertainties were found to be smaller for MonoCT compared to SECT.

While the small patient cohort used in this study was sufficient as a proof-of-concept for use of tissue-specific uncertainties on a voxel-wise basis, evaluations based on a larger patient cohort is required to make real assessments on its feasibility as an alternative to the fixed uncertainties. Our patient cases were mainly dominated by medium-density tissues (up to 98 %), and a more thorough examination should include patient cases with higher percentages of low- and high-density tissues as well. Additionally, employment of robust optimization and evaluation is needed to assess the actual dosimetric differences of applying the different uncertainty types. Plan optimization was intentionally performed without robust optimization in this study to clearly see the effect of the two different SPR uncertainty approaches (tissue-specific vs fixed). Also, it is currently not possible to apply tissue-specific SPR uncertainties directly in robust optimization in any commercial treatment planning systems, but this may be accomplished by including the nominal SPR maps and the SPR maps with uncertainties added/subtracted in a 4D robust optimization where several CT images (here three) can be included in the robust optimization [[19], [20]].

In conclusion, we have identified a potential gain of applying tissue-specific uncertainties on a voxel-wise basis. However, a single fixed uncertainty may still be sufficient to capture the tissue-specific uncertainties in some cases, but with a magnitude which depends on the tissue composition of the treatment site. However, further development of the study, in terms of a larger patient cohort and use of robust optimization, are required to effectively evaluate the potential of applying tissue-specific range uncertainties on a voxel-wise basis in clinical practice.

## Declaration of Competing Interest

The authors declare that they have no known competing financial interests or personal relationships that could have appeared to influence the work reported in this paper.
