# Supplementary material for: Tissue-specific range uncertainty estimation in proton therapy
Source: Phys Imaging Radiat Oncol. 2023 Apr 22;26:100441. doi: 10.1016/j.phro.2023.100441 (PMC10173296; doi:10.1016/j.phro.2023.100441)
Supplement: Supplementary data 1 [file mmc1.pdf]

Supplementary Materials

Supplementary Material A:

Stoichiometric calibration:

This section briefly outlines how the stopping power ratio (SPR) values and CT numbers, used for calibration of the stoichiometric conversion curve, were calculated and estimated, respectively. For a thorough description, the reader if referred to the original paper by Schneider *et al.* [1].

The theoretical SPR values were calculated with the Bethe equation, which can be approximated by [1]:

$$S = \rho_e \cdot \frac{\ln(2m_e c^2) + \ln\left(\frac{\beta^2}{1-\beta^2}\right) - \beta^2 - \ln(I_m)}{\ln(2m_e c^2) + \ln\left(\frac{\beta^2}{1-\beta^2}\right) - \beta^2 - \ln(I_w)} \quad \text{(A.1)}$$

where ρ_e is the ratio of electron density of the medium and of water (the so-called relative electron density; RED), β is the proton velocity relatively to the speed of light, c, and m_e is the mass of the electron. $\ln(I_m)$ and $\ln(I_w)$ are the natural logarithm of the mean excitation energy (MEE) of the medium and water, respectively. Under the assumption that the kinetic energy of the protons is unchanged as the protons traverse through the tissues, the only dependences of SPR in Eq. (A.1) are the RED and the MEE of the medium. The RED can be directly calculated from the elemental composition of the compound, while the MEE of a compound can be approximated by the Bragg additivity rule [1]. In this study, a fixed proton kinetic energy of 200 MeV was used in consistency with Yang *et al.* [2].

The CT numbers in Hounsfield Units (HU) are defined through the ratio of energy-averaged attenuation coefficients, $\langle\mu\rangle$, for the medium and water. Rutherford *et al.* [3] suggested that $\langle\mu\rangle$ could be modeled by a parametrization with three CT scanner-specific parameters:

$$\langle\mu\rangle = \rho \cdot N_g(Z, A) \cdot \left(K^{ph} \cdot \left[\frac{N_g^i}{N_g} \cdot Z_i^{3.62}\right]^{1/3.62} + K^{coh} \cdot \left[\frac{N_g^i}{N_g} \cdot Z_i^{3.62}\right]^{1/1.86} + K^{KN}\right) \quad \text{(A.2)}$$

where ρ is the mass density, K^{ph}, K^{coh} and K^{KN} are CT scanner-specific parameters describing the energy spectrum, and N_g is the number of electrons per unit volume of the mixture given by:

$$N_g = \sum_i N_g^i = N_A \sum_i \frac{w_i Z_i}{A_i} \quad \text{(A.3)}$$

where N_A is Avogadro's number, w_i is the weight fraction of element i, Z_i is the atomic number of element i, and A_i is the atomic mass of element i. The three scanner specific parameters were determined by minimizing the difference between the estimated CT numbers and measured CT numbers for a set of materials of known elemental composition using a nonlinear least square regression. In this study, the scanner-specific parameters were determined using the fourteen tissue-equivalent phantom inserts (see section CT scanner protocol below). After determining the scanner-specific parameters, the estimated CT numbers were computed for a set of 71 tabulated human tissues [4,5]. The conversion curve was then created by fitting the estimated CT numbers to the calculated SPR values of the tabulated human tissues. The resultant conversion curve was divided into three piece-wise linear fits (one for each of the three tissue groups, low-, medium-, and high-density) and two connecting segments.

CT scanner protocol:

In this study, the Advanced Electron Density Phantom (Gammex, Sun Nuclear, Middleton, WI, USA) was scanned with a SOMATOM Definition Edge CT scanner (Siemens Healthineers, Forchheim, Germany) in dual-energy CT (DECT) twin-beam (TB) mode. Both the head part (round, 20 cm diameter) and body part (elliptical, 40x30 cm) of the phantom were scanned, using a cerebrum and abdomen protocol, respectively. Scanning parameters included: pitch: 0.45, rotation time: 1.0 s, collimation width: 2.0 mm, $CTDI_{vol(16cm)}$: 40.0 mGy (head) / $CTDI_{vol(32cm)}$: 20.2 mGy (body). The images were reconstructed with the Qr40 kernel with beam hardening correction for bone (iBHC bone), applying iterative reconstruction (ADMIRE, strength level 3). The resultant DECT images were used to create 90 keV MonoCT images applying the MonoPlus algorithm with the Siemens *syngo*.via software. A total of fourteen tissue-equivalent phantom inserts (ranging from lung to cortical bone) were scanned and used for determining the three scanner-specific parameters of the stoichiometric conversion curve (configuration 1 and 3; Figure A1) and for SPR uncertainty estimation (configuration 1-3; Figure A1). For each insert, the mean CT number and standard deviation were calculated from the CT numbers sampled from ten central slides in a circular region-of-interest (diameter = 1.8 cm) in the middle of the insert (Table A1).

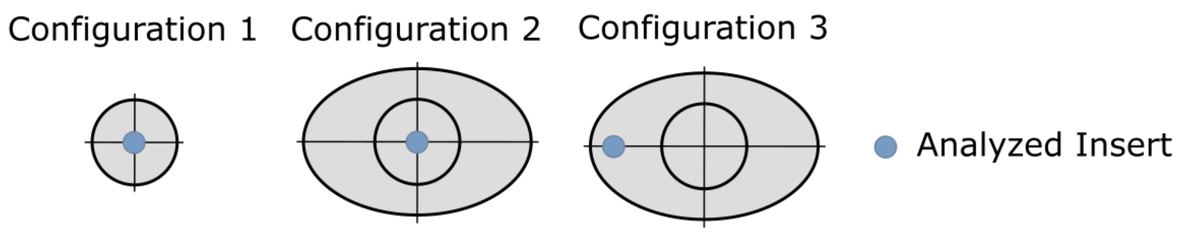

Figure A1: Illustration of the three measurement configurations examined in this study. The figure is inspired by [6].

Table A1: Mean CT number and standard deviation in HU of the fourteen tissue-equivalent phantom inserts used for calibration of the stoichiometric calibration method (configuration 1) and for the SPR uncertainty analysis (configuration 1-3).

Tissue-equivalent Phantom Inserts			
**Insert Name**	**Mean CT number (standard deviation) in configuration 1**	**Mean CT number (standard deviation) in configuration 2**	**Mean CT number (standard deviation) in configuration 3**
**Lung (LN-300)**	-700 (35)	-684 (22)	-694 (21)
**Lung (LN-450)**	-529 (14)	-518 (30)	-522 (23)
**HE General Adipose**	-58 (5)	-49 (11)	-56 (9)
**HE Breast 50:50**	-30 (5)	-23 (12)	-29 (9)
**CT HE Solid Water**	4 (5)	7 (10)	4 (9)
**HE Brain**	31 (5)	35 (10)	32 (10)
**HE Liver**	60 (5)	63 (10)	59 (9)
**Inner Bone**	178 (5)	175 (15)	171 (13)
**B-200 Bone**	188 (6)	187 (14)	181 (13)
**HE Inner Bone**	253 (5)	251 (14)	247 (10)
**30% $CaCO_3$**	396 (6)	395 (14)	391 (11)
**50% $CaCO_3$**	705 (7)	688 (17)	688 (12)
**Cortical Bone**	1065 (8)	1032 (20)	1037 (15)
**HE Cortical Bone**	1185 (9)	1047 (20)	1148 (16)

Supplementary Material B:

CT imaging uncertainties:

The CT imaging uncertainties are caused by CT image noise and the beam hardening effect due to patient size and tissue positioning within the patient. The variation in CT numbers due to noise, σH_{noise}, was estimated as the standard deviation of the CT numbers inside the volume-of-interest of the inserts measured in middle of the head part of the phantom (configuration 1 in Figure A1). Note that very large standard deviations were found for the two lung inserts (Table A1). This was assumed to be due to the inhomogeneity of these two inserts [7]. For this reason, the average standard deviation of the medium-density tissues was used as the σH_{noise} for the low-density tissues, resulting in 5 HU.

The CT number variation due to the beam hardening effect was evaluated in two ways. The patient size dependency was quantified as the CT number difference between inserts scanned in the middle of the head and body phantom (σH_{size}; configuration 1 and 3 in Figure A1). While the tissue position dependency was quantified as the CT number difference between inserts in the center and periphery of the body phantom (σH_{pos}; configuration 2 and 3 in Figure A1).

The CT number variation, σH, was translated into unsigned uncertainty in SPR, U_{uns}, using the slopes of the conversion curves, α (see Eq. (1) in the main article). For the estimation of the unsigned uncertainty in SPR due to CT imaging noise and patient size, the slope of the conversion curve calibrated with the head phantom was used. For the estimation of the unsigned uncertainty in SPR due to tissue positioning, the slope of the conversion curve calibrated with the body phantom was used. Relative uncertainties in SPR (U_{rel}) were obtained from the unsigned uncertainties in SPR (U_{uns}) with respect to the SPR of the inserts scanned in configuration 1 (configuration 3 for the tissue positioning) in Figure A1 using Eq. (2) in the main article. The total CT imaging uncertainty was obtained using the Guide to the expression of Uncertainty in Measurement (GUM) [8]. According to the GUM, the individual uncertainty contributions can be propagated to a total uncertainty by:

$$\sigma_{tot} = \sqrt{\sum_i (c_i \cdot U_i)^2} \quad \text{(B.1)}$$

where U_i are the individual uncertainty contributions and c_i are divisors that depends on the probability density function (PDF) of the corresponding uncertainty contribution. However, as all three contributions to the CT imaging uncertainties were estimated based on limited amount of data, a good assessment of the PDFs could not be made. The PDFs were therefore simply assumed to be normal for all three contributions, as the normal distribution entails the highest scaling factor, $c_i = 1$ (thereby ensuring not underestimating the total CT imaging uncertainty). Additionally, independence between the three contributions were assumed for simplicity (no covariance) as done in previous studies [2,9], and the total CT imaging uncertainty was therefore obtained by:

$$U_{uns/rel}^{img} = \sqrt{\left(U_{uns/rel}^{noise}\right)^2 + \left(U_{uns/rel}^{size}\right)^2 + \left(U_{uns/rel}^{pos}\right)^2} \quad \text{(B.2)}$$

where $U_{uns}^{noise}, U_{uns}^{size}$ and U_{uns}^{pos} are the unsigned SPR uncertainties from the three contributions (noise, size and positioning), respectively, and $U_{rel}^{noise}, U_{rel}^{size}$ and U_{rel}^{pos} are the relative SPR uncertainties from the three contributions, respectively. The unsigned and relative SPR uncertainties were first calculated individually for each tissue insert. To obtain the SPR uncertainties for each specific tissue group (low-, medium- and high-density tissues), the unsigned and relative SPR uncertainties were averaged over the inserts within each of these three tissue groups.

CT number estimation uncertainties:

The uncertainty in the estimation of CT numbers using the parametrization, Eq. (A.2), was assessed as the unsigned difference between the measured and the estimated CT numbers of the fourteen inserts, $\sigma H = |H_{meas} - H_{est}|$. The corresponding unsigned and relative uncertainties in SPR were calculated from Eq. (1) and Eq. (2) in the main article, respectively. The mean values for this uncertainty were calculated over the inserts belonging to each of the three tissue groups.

SPR estimation uncertainties:

To assess the uncertainty in SPR estimation for biological tissues, the deviations between the theoretical SPR values and the estimated SPR values were computed for the 71 tabulated human tissues. Prior to the calculation and estimation of the SPR, a series of changes were introduced in the densities and elemental compositions to account for the fact that real biological tissues may be different from tabulated human tissues. Following the same procedure as proposed by Yang *et al.* [2], three changes were introduce: i) the densities were varied for all tissues; ii) the hydrogen weight fraction were changed for low- and medium-density materials; and iii) the calcium weight fraction were changed for high-density materials. To maintain a total elemental weight fraction of 100% for each tissue, a reduction of the weight fraction of hydrogen or calcium were counter-acted by an increase of the weight fraction of oxygen or carbon, and vice versa.

In this study, a total of 3000 tissues were generated for each of the 71 tissues; 1000 with changed density, 1000 with changes to the weight fraction of hydrogen/calcium compensated by weight fraction changes of carbon, and 1000 with changes to the weight fraction of hydrogen/calcium compensated by weight fraction changes of oxygen. It was assumed that the elemental composition and density could be changed independently as assumed in previous studies [2,10,11]. After each change, the relative and absolute difference between the theoretical SPR values, Eq. (A.1), and estimated SPR values (using the stoichiometric conversion curve) were calculated for each tissue. The average of the 1000 differences for each change were then calculated and noted as the SPR estimation uncertainty for the specific change and tissue. Finally, the average of these numbers was calculated for tissues belonging to each of the three tissue groups for each change. This entire process was repeated 100 times to eliminate the statistical influence of the approach and the average of the results was calculated. For each tissue group, the largest difference when changing the densities or elemental percentages was taken as the SPR estimation uncertainty. For the full description of this category of uncertainties, the reader is referred to the original paper [2].

Total tissue-specific uncertainties:

The total tissue-specific SPR uncertainties ($\mathrm{U}^{\mathrm{tot}}$) were obtained from the five uncertainty contributions (CT imaging ($\mathrm{U}^{\mathrm{img}}$), CT number estimation (U^{H}), SPR estimation ($\mathrm{U}^{\mathrm{stoi}}$), mean excitation energy (MEE; $\mathrm{U}^{\mathrm{MEE}}$), and ignorance of the energy dependence on SPR (U^{E})) using the GUM. As for the CT imaging uncertainties, the five uncertainties contributions were assumed to be independent for simplicity [2,9]. The PDF of the CT estimation uncertainty was assumed to be normal, due to the limited amount of data used in the estimation of the uncertainty contribution, while the PDF of the SPR estimation uncertainty was normal due to the choice of sampling the changed tissues from normal distributions. The two model independent uncertainties were quantified assuming normal distributions as well [12]. Hence, the total unsigned/relative total tissue-specific uncertainties were obtained from the five uncertainty contributions by:

$$\mathrm{U}_{\mathrm{uns/rel}}^{\mathrm{tot}} = \sqrt{\left(\mathrm{U}_{\mathrm{uns/rel}}^{\mathrm{img}}\right)^2 + \left(\mathrm{U}_{\mathrm{uns/rel}}^{\mathrm{H}}\right)^2 + \left(\mathrm{U}_{\mathrm{uns/rel}}^{\mathrm{stoi}}\right)^2 + \left(\mathrm{U}_{\mathrm{uns/rel}}^{\mathrm{MEE}}\right)^2 + \left(\mathrm{U}_{\mathrm{uns/rel}}^{\mathrm{E}}\right)^2} \quad \text{(B.3)}$$

Supplementary Material C:

Patients:

Four patients were used in the evaluation of the tissue-specific uncertainties: a brain cancer patient, a lymphoma cancer patient, and two liver cancer patients. The two liver patients and the lymphoma patient were scanned using 120 kVp single-energy CT (SECT; voxel size of 0.97x0.97x2mm^3), while the brain patient was scanned using DECT in twin-beam (TB) mode (voxel size of 0.63x0.63x1.5mm^3). For the brain patient, Siemens *syngo*.via software was used to create a 90 keV monoenergetic CT (MonoCT) image set applying the MonoPlus algorithm based on the DECT images. All CT scans were reconstructed with the Qr40 kernel with beam hardening correction for bone (iBHC bone). Prescribed dose, number of fractions, beam and couch angles and tissue percentages for each treatment are shown in Table C1.

Table C1: Prescribed dose, number of fractions, and beam and couch angles used to create the treatment plan for the four patients. The last column shows the percentage of each tissue type within the beam path (D>0 Gy in the nominal plan; the tissue type of a CT voxel is classified in terms of the CT number: low-density: $-900 < \mathrm{H} \leq -228\ \mathrm{HU}$; medium-density: $-228\ \mathrm{HU} < \mathrm{H} \leq\ 154\ \mathrm{HU}$ and high-density: $\mathrm{H} > 154\ \mathrm{HU}$).

	**Prescribed dose (Gy)**	**Number of fractions**	**Beam (and couch) angles (° degrees)**	**Tissue percentages** (low, medium, high)
**Lymphoma cancer patient**	19.8	11	20, 350<br>(0, 0)	18%, 78%, 4%
**Brain cancer patient**	59.4	33	82, 225, 310<br>(0, 0, 280)	8%, 78%, 14%
**Cranial liver cancer patient**	58	15	250, 280, 310<br>(0, 0, 0)	12%, 87%, 1%
**Caudal liver cancer Patient**	58	15	170, 270, 310<br>(0, 0, 0)	1%, 98%, 1%

References

[1] Schneider U, Pedroni E, Lomax A. The calibration of CT Hounsfield units for radiotherapy treatment planning. Phys Med Biol 1996;41:111–24. https://doi.org/10.1088/0031-9155/41/1/009

[2] Yang M, Zhu XR, Park PC, Titt U, Mohan R, Virshup G, et al. Comprehensive analysis of proton range uncertainties related to patient stopping-power-ratio estimation using the stoichiometric calibration. Phys Med Biol 2012;57:4095–115. https://doi.org/10.1088/0031-9155/57/13/4095

[3] Rutherford RA, Pullan BR, Isherwood I. Measurement of effective atomic number and electron density using an EMI scanner. Neuroradiology 1976;11:15–21. https://doi.org/10.1007/BF00327253

[4] White DR, Woodard HQ, Hammond SM. Average soft-tissue and bone models for use in radiation dosimetry. Br J Radiol 1987;60:907–13. https://doi.org/10.1259/0007-1285-60-717-907

[5] Woodard HQ, White DR. The composition of body tissues. Br J Radiol 1986;59:1209–18. https://doi.org/10.1259/0007-1285-59-708-1209

[6] Randers P, Jensen MF, Taasti VT. Commissioning mono-energetic CT images for optimal proton dose calculations using TwinBeam scans, Italy: 2019. https://doi.org/10.1016/s0167-8140(19)32472-7

[7] Hudobivnik N, Schwarz F, Johnson T, Agolli L, Dedes G, Tessonnier T, et al. Comparison of proton therapy treatment planning for head tumors with a pencil beam algorithm on dual and single energy CT images. Med Phys 2016;43:495. https://doi.org/10.1118/1.4939106

[8] JCGM 2008 Evaluation of Measurement Data—Guide to the Expression of Uncertainty in Measurement (GUM) (Report 100:2008, BIPM) (Sèvres: Joint Committee for Guides in Metrology/WG 1)

[9] Je E, Lee HH, Duan X, Li B, Jia X, Yang M. Optimal energy selection for proton stopping-power-ratio estimation using dual-energy CT-based monoenergetic imaging. Phys Med Biol 2019;64:195015. https://doi.org/10.1088/1361-6560/ab3dec

[10] Yang M, Virshup G, Clayton J, Zhu XR, Mohan R, Dong L. Theoretical variance analysis of single- and dual-energy computed tomography methods for calculating proton stopping power ratios of biological tissues. Phys Med Biol 2010;55:1343–62. https://doi.org/10.1088/0031-9155/55/5/006

[11] Taasti VT, Petersen JBB, Muren LP, Thygesen J, Hansen DC. A robust empirical parametrization of proton stopping power using dual energy CT. Med Phys 2016;43:5547. https://doi.org/10.1118/1.4962934

[12] Peters N, Wohlfahrt P, Hofmann C, Möhler C, Menkel S, Tschiche M, et al. Reduction of clinical safety margins in proton therapy enabled by the clinical implementation of dual-energy CT for direct stopping-power prediction. Radiother Oncol 2021;166:71-8. https://doi.org/10.1016/j.radonc.2021.11.002
